# Inhibition of energy metabolism in macrophages to block MPS for enhancing the chemotherapy efficacy

**DOI:** 10.3389/fbioe.2025.1549101

**Published:** 2025-04-04

**Authors:** Li Bin, Linlin Huang, Aiyu Chen, Yinyi Yang, Yanmei Zheng, Hanwen Zhang, Qinfang Zhang, Jiahui Zheng, Meiting Qiu, Xiajin Li, Yangbo Tan

**Affiliations:** ^1^ Department of Medical College, Guangxi University of Science and Technology, Liuzhou, China; ^2^ Laboratory animal Center, Liuzhou People’s Hospital, Liuzhou, Guangxi, China

**Keywords:** glucose transporter 1, energy metabolism, MPS, metastasis, chemotherapy

## Abstract

Various biological barriers hinder the effective use of administered nanoparticles, with the mononuclear phagocyte system (MPS) being a major obstacle to their *in vivo* efficacy. Glucose metabolism is an important factor for macrophages to perform MPS clearance *in vivo*. In this study, energy metabolism-blocking nanoparticles PEG-S-S-PLA@RGD @Dox@BAY876 (RPDB NPs) were developed to change drug distribution in the body, improving the efficacy of chemotherapy. First, BAY876 showed an excellent inhibition effects on macrophage energy metabolism *in vitro*. This inhibitory behavior of energy metabolism reduced the aggregation of nanoparticles in macrophages. Similarly, the migration capacity of macrophages was also limited by reduced energy metabolism. Second, the fluorescence distribution in the mice also showed that the fluorescence intensity of RPDB NPs in the liver was about 40% of that of RPD NPs, suggesting that reducing energy metabolism helps to downregulate the uptake of mononuclear phagocytic cell (MPS), and change the distribution of the drug *in vivo*. Furthermore, anti-tumor effects of RPDB NPs were evaluated both *in vivo* and *in vitro*. *In vivo*, RPDB nanomicelles inhibited breast cancer by up to 68.3%, higher than other administration groups. Moreover, the pathological section of tumor exhibited a significantly greater increase in cell apoptosis in RPDB NPs group. Hence, inhibition of macrophage energy metabolism is a promising approach to eliminate MPS effects, while also opening up a new window for the effective inhibition of tumors development and metastasis.

## 1 Introduction

Nanomedicine has emerged as a promising field in biomedicine over the past few decades, owing to the exceptional pharmacokinetics and high-contrast imaging capabilities of nanotherapeutics and diagnostic agents (Wang et al., 2011; Li and Lane, 2019). However, only 0.7% injected doses of nanoparticles (NPs) can reach solid tumors, which limits the therapeutic effect (Poon et al., 2020). The delivery inefficiency arises from the mononuclear phagocyte system (also called the reticuloendothelial system, RES), which was capable of rapidly recognizing and engulfing exogenous nanoparticles (Liu et al., 2017; Yang et al., 2016; Wan et al., 2020). Under the influence of MPS, nanoparticles tend to accumulate in normal tissues, particularly in the liver, which may lead to potential adverse effects (Mirkasymov et al., 2021). Therefore, efficient strategies for regulating the mononuclear phagocyte system (MPS) are highly promising for improving tumor accumulation (Mirkasymov et al., 2022).

Numerous strategies have been devised and implemented to alleviate the influence of MPS (Mirkasymov et al., 2022). Obstructing the recognition of macrophages and nanoparticles can be achieved through various methods, such as reducing the immunogenicity of nanoparticles by modifying their surface with polymers or camouflaging them with biomimetic materials and “do not eat me” signal molecules to evade detection by macrophages (Yang et al., 2022; Wang et al., 2019; Belhadj et al., 2020). Blocking macrophages through saturation is a promising tactic that endows nanoparticles with several benefits, including an extended duration of blood circulation (Ngo et al., 2022; Li et al., 2023; Cheng et al., 2021; Ouyang et al., 2020). For instance, a considerable quantity of nanoparticles was phagocytosed by macrophages *in vivo*, surpassing the RES threshold and penetrating the immune barrier, thereby prolonging the circulation time of blood and facilitating accumulation in tumors (Ouyang et al., 2020). Furthermore, the blocking efficiency of RES showed a positive correlation with the administered dosage, and macrophage saturation occurred within 1.5 h following the injection of one trillion nanoparticles (Ouyang et al., 2020). However, the burden of eliminating nanoparticles in normal tissues was significantly increased at high dosages of administration, potentially leading to inflammation in the aggregation area.

The strategy of eliminating macrophages to obstruct the mononuclear phagocyte system (MPS) has attracted significant attention. These agents were taken up by MPS, resulting in macrophage blockade followed by an extension of the circulation time of nanoparticles *in vivo*. This is primarily attributed to the impairment of MPS-mediated immune clearance of exogenous substances, particularly the Kupffer cells in the liver which constitute a pivotal component of MPS (Tavares et al., 2017). The drug-induced blockage phenomenon *in vivo* is transient, and hepatic macrophages typically recover promptly (Hao et al., 2018). For instance, the administration of dextran sulfate 500, a polysaccharide compound, at a dosage of 50 mg/kg significantly reduced the level of liposomes in the liver and disappeared within 48 h (Patel et al., 1983). The clodronate was encapsulated in nanoparticles to achieve macrophage depletion, resulting in a remarkable 80% increase in the rate of tumor inhibition (Hao et al., 2018). In addition, liposomal encapsulation of methylpalmitate and gadolinium chloride demonstrated significant macrophage clearance effects by regulating the ATP levels, respectively, similar to clodronate (Yamada et al., 2003). Thus, the main metabolic process of numerous MPS blockers like clodronate used frequently for macrophage scavenging is their conversion through hydrolysis into an analog that hinders ATP synthesis. However, the complete elimination of macrophages will affect the body’s immune function and even cause damage to the body.

Similarly, regulating glucose uptake to inhibit the energy metabolism of macrophages and reduce ATP production has become a feasible strategy for the drug development (Curi et al., 2017; Sakamoto et al., 2011; Jacobs et al., 2008). Glucose plays a crucial role in maintaining cellular activity and promoting nanoparticles phagocytosis by cells (Zhao et al., 2011; Bonamy et al., 2023; Venturelli et al., 2016). Glucose transporters, which are responsible for glucose uptake into cells, exhibit high levels of expression in macrophages, particularly in M2-like tumor-associated macrophages (Shi et al., 2022). Considering the propensity of nanoparticles to accumulate in the liver, with macrophages influencing up to 70% of them, it was hypothesized that the energy metabolism in macrophages may play a pivotal role in particle up-taken (Venturelli et al., 2016; Shi et al., 2022). As a proof of concept, the inhibition of glucose transporters 1 (GLUT1) protein in macrophages by a GLUT1 inhibitor was employed to curtail tricarboxylic acid cycle (TCA cycle) energy metabolism. Subsequently, MPS blockade was implemented to enhance nanoparticles aggregation within tumors. According to previous reports, BAY876, a GLUTs family inhibitor with a particular affinity for GLUT1, has demonstrated remarkable antitumor efficacy by reducing glucose levels and limiting glycolysis (Zhao et al., 2011; Chen et al., 2023).

Herein, tailor-made self-assembled nano-micelles of PEG-SS-PLA polymers containing disulfide bonds were used to improve the distribution of nanoparticles *in vivo* by interfering with the energy metabolism of macrophages in the liver. In addition, RGD peptides were introduced to prevent off-target phenomena and facilitate the investigation of drug distribution *in vivo*. Under the influence of MPS, the nanomicelles were internalized by liver macrophages and subsequently released BAY876 in lysosomes to inhibit GLUTs and ATP production. This macrophage blockade strategies reduced the persistent accumulation of nanomicelles in the liver. Therefore, this system effectively prolonged blood circulation time and enhanced the tumor accumulation of nanoparticles. Meanwhile, the utilization of nano-micelles integrated with macrophage elimination and chemotherapy strategy further enhanced the anti-tumor efficacy.

## 2 Materials and methods

### 2.1 Materials

All reagents without further purification were acquired from commercial corporations in this study. The paraformaldehyde (4%), phosphate buffer (PBS, pH7.4), doxorubicin (Dox), thiazolyl blue (MTT), and dimethyl sulfoxide (DMSO) were bought from Servicebio (Wuhan, China). The BAY-876 was obtained from MCE China (Shanghai). The Cy5.5-COOH was purchased from Aladdin (Shanghai, China). The RPMI-1640 complete medium and fetal serum (FBS) was purchased from KeyGen Biotech Co., Ltd. (Nanjing, China). Hoechst 33342, the nucleus-specific dye utilized in this study, was procured from Beyotime Biotechnology Ltd. (Shanghai, China). In addition, the GSH assay kit and Glucose assay kit and ATP assay kit were also acquired from Beyotime Biotechnology Ltd. (Shanghai, China). The mouse-derived 4T1 cancer cell was obtained from the Shanghai Institute of Cells (Shanghai, China). The polymer PEG2k-S-S-PLA2k (Polyethylene glycol-S-S-polylactic acid) was obtained from Chongqing Yusi Biotech Co. Ltd. (Chongqing, China). The SPF female mice (Balb/c, 5 weeks old) were purchased from SPF Biotechnology Co., Ltd. (Beijing, China) and maintained in a pathogen-free environment. The experiments protocols were reviewed and approved by Institutional Animal Care and Use Committee of Liuzhou People`s Hospital (Liuzhou, China, IACUC No. Approved:LRYIACUC2023001). All experiments were performed with deionized water (Persee, 18.2 MΩ cm^−1^).

### 2.2 Preparation of nano-micelle complexes

As previously described, a solution containing EDC and NHS was used to activate the carboxyl group of the target peptide (RGD) under pH5.8 for 30 min (molar ratio 1.2:1.2:1). Then, the NH2-PEG2k-PLA2k were added and incubated for 12 h. After that, the RGD-modified polymer (RP) were collected by centrifugation at 15000 RPM for 15 min. Similarly, Cy5.5-COOH reacted with EDC and NHS for 30 min under dark before adding RP. After 12 h, Cy5.5-labeled RP was centrifuged, purified, and stored at 4°C. Last, the nano-micelle complexes were prepared using the oil-in-water emulsion solvent diffusion method. To prepare the solution, 2.8 mg of Dox, 3.2 mg of BAY876, and 20.1 mg of the RGD-modified polymer (RP) were dissolved in 10 mL of chloroform. Removed the organic solvent by rotary evaporation in a 50°C water bath and collected the nano-micelle complexes (RPDB NPs) in an equal volume of PBS phosphate buffer solution. Encapsulation percentage=(1-The amount of free drug/The total amount of drug)*100%.

### 2.3 Characterization

To observe the morphology of RPDB NPs, a transmission electron microscope (HC-1, Hitachi, Japan) was used. The particle size and polydispersity were effectively determined by the dynamic light scattering (DLS) technique, which utilized Malvern Instruments. Then, the UV-2600 UV-vis spectrophotometer (Shimadzu, Japan) was successfully used for detecting absorbance and concentration. To study the release behavior of RPDB NPs in tumors, these particles were suspended in 1 mL of PBS with GSH (4 mM). In addition, the release behavior under pH 5.0 was further examined. Finally, the stability of nanoparticles in fetal bovine serum was monitored by DLS for 20 days under 37°C.

### 2.4 Evaluation of glucose uptake in macrophages

Macrophage RAW264.7 inoculated with 10% (v/v) FBS and DMEM incomplete medium under a humidified atmosphere (37°C) with 5% CO_2_. When the density of macrophage cells reached 80%, the culture medium was discarded, and the dishes were washed three times with PBS phosphate buffer. The cells were digested by trypsin-EDTA and subsequently collected in a sterile centrifuge tube. After centrifugation, the macrophage cells were subcultured in a 6-well plate with 8 × 10^4^ cells per well. Then, the cells were grouped as follows: RPD (50 μg mL^−1^, according to the Dox concentration + LG (low glucose medium) group, RPD (50 μg mL^−1^, according to the Dox concentration + HG (high glucose medium) group, RPDB(50 μg mL^−1^, according to the Dox concentration + HG (high glucose medium) group and PBS group. The PBS group was incubated with high glucose (HG) DMEM. 24 h later, the dye was marked under fluorescence microscopy with different excitation wavelength (Hoechst 33342:350 nm, Dox: 488 nm).

### 2.5 Cytotoxicity assay

To evaluate cytotoxicity *in vitro*, 4T1 cells (5 × 10^3^ cells per well) were inoculated into each well of a 96-well plate. After 12 h, the cells were grouped as follows: P, Dox (50 μg mL^−1^), RPD (50 μg mL^−1^, according to the Dox concentration + LG (low glucose medium) group, RPD (50 μg mL^−1^, according to the Dox concentration + HG (high glucose medium) group, RPDB (50 μg mL^−1^, according to the Dox concentration + HG (high glucose medium) group and PBS group. The PBS group was incubated with high glucose (HG) DMEM. Following a 24-h co-culture of nano-micelle complexes with cells, the living cell count was determined using MTT reagent. Then, the medium in each well was discarded, and 150 μL dimethyl sulfoxide (DMSO) was used to dissolve the crystals. Finally, the absorbance of each well at 490 nm was measured by the continuous wavelength multifunctional microplate reader (Victor Nivo 3S, United States). The cell viability (%) of nano-micelle complexes was calculated using the following method: OD treatment/OD control × 100%.

### 2.6 Glucose and ATP levels *in vitro*

To verify the effect of the GLUT1 inhibitor (BAY876), the cellular glucose uptake and ATP level was measured *in vitro*. Briefly, macrophage RAW264.7 and 4T1 cells were pre-cultured in 6-well plates (8 × 10^4^ cells per well, 2 mL medium), respectively. Overnight, these cells were grouped and treated with the following samples: RPD (50 μg mL^−1^, according to the Dox concentration + LG (low glucose medium) group, RPD (50 μg mL^−1^, according to the Dox concentration + HG (high glucose medium) group, RPDB(50 μg mL^−1^, according to the Dox concentration + HG (high glucose medium) group and PBS group. The PBS group was incubated with high glucose (HG) DMEM. After 24 h, all cells were treated with lysis buffer (containing Triton-X-100) and broken using ultrasonic (150 W, 30 s). After cracking, centrifuged at 4°C (12,000 g) for 5 min and collected supernatant. (1) ATP level: Added 100 μL of ATP test fluid to the plate. After 3 min at room temperature, added 20 μL of sample to each well, and tested with a multifunctional microplate reader. (2) The glucose level:Taken 5 µL sample to PCR tube and added 185 µL glucose assay reagent to make the final volume 190 µL. Placed on a PCR apparatus and heated at 95°C for 8 min, then cooled down to 4°C. Then 180 µL of liquid was sucked into the 96-well plate and absorbance was measured at 630 nm. The glucose concentration and ATP levels in the sample was calculated from the standard curve, respectively.

### 2.7 Detection of GSH level *in vitro*

4T1 cells were incubated with RPMI-1640 medium (including 10% (v/v) serum (FBS), 100 U/mL penicillin, and 100 μg/mL streptomycin). After overnight incubate, the samples were treated with cells in the following order: RPD (50 μg mL^−1^, according to the Dox concentration + LG (low glucose medium) group, RPD (50 μg mL^−1^, according to the Dox concentration + HG (high glucose medium) group, RPDB(50 μg mL^−1^, according to the Dox concentration + HG (high glucose medium) group and PBS group. The PBS group was incubated with high glucose (HG) DMEM. Then, the procedure was performed as described above. The GSH level was measured using an assay kit and the absorbance of GSH was recorded with a microplate reader.

### 2.8 Scratch assay *in vitro*

To assess the impact of a GLUT1 inhibitor on macrophage cell migration, an *in vitro* scratch assay was employed as a direct and cost-effective method. As previously described, RAW264.7 macrophage cells were seeded in a 6-well plate with DMEM (high glucose) containing 10% FBS for 12 h. Then, monolayer cells in a 6-well plate were scratched with a sterile pipette and washed with phosphate buffer to remove cell debris. After being cleaned, the samples were treated with macrophage cells for 24 h. These wounds were imaged using an inverted microscope, and the healing of wounds was evaluated by software ImageJ. The migration rate = (scratch width at 0 h - scratch width at 48 h)/scratch width at 0 h *100%

### 2.9 Tumor-bearing mice models

All healthy mice were purchased and housed for a week. Then, the 4T1 cells (5 × 10^6^ cells/mouse, 100 μL) were injected into the right forelimb of each mouse to establish a solid tumor model. Thereafter, the mouse tumor volume was observed and measured every day using the formula length × width^2^ × 0.5 to assess tumor volume.

### 2.10 *In vivo* distribution of nano-micelle complexes

To investigate the *in vivo* distribution of nano-micelle complexes, Cy5.5-labeled RPDB NPs and RPD NPs (100 μL, 2 mg/mL in PBS phosphate buffer) were tracked in tumor-bearing mice using IVIS Spectrum (Perkin Elmer, America). The excitation and emission wavelengths of Cy5.5 are 673 nm and 707 nm, respectively. These mice were then euthanized, the fluorescence intensity of their tumors was measured at various time points for 24 h, and the images were analyzed with ImageJ.

### 2.11 *In vivo* antitumor efficacy

After 5 days, when the tumor volume of mice (n ≥ 5) reached approximately 100 mm^3^, intravenous injections of PBS, Dox, RPD NPs, PDB NPs, and RPDB NPs were administered at a dose of 100 µL per mouse with a concentration of 5 mg/kg based on Dox concentration to the mice bearing the 4T1 tumors. Take an injection every 2 days. The body weight and tumor volume were measured and calculated to evaluate antitumor efficacy. After 13 days, the mice were humanely sacrificed, and their primary tumors, major organs, and blood samples were collected for further analysis.

### 2.12 Tumor metastasis model

In this anti-tumor metastasis, the mice were divided into the following groups: P1group (Each mouse was injected with 5 × 10^6^ live cells), P2 group (Each mouse was injected with 5 × 10^6^ dead cells), P3 (Each mouse was injected with 5 × 10^6^ dead cells and 5 × 10^6^ live cells), RPD NPs group (Each mouse was injected with 5 × 10^6^ dead cells and 5 × 10^6^ live cells) and RPDB NPs group (Each mouse was injected with 5 × 10^6^ dead cells and 5 × 10^6^ live cells). Then, 4T1 breast cancer cells were cultured and injected into mice tail vein for construct tumor metastasis model. RPD NPs was subsequently injected into RPD NPs group, RPDB NPs was subsequently injected into RPDB NPs group. After 14 days, the lung of mice were dissected to observe tumor metastasis.

### 2.13 Histopathology and routine blood analysis

In order to further investigate the effect and biosafety of nano-micelle complexes, major organs, and tumors were fixed in 4% paraformaldehyde at 4°C for 12 h. These slices were then prepared, including dehydration, embedding, and slicing, followed by dewaxing. Immediately, H&E staining and immunohistochemical analysis were performed, and all sections were observed under an inverted microscope. The blood samples of mice were collected for routine hematological analysis.

### 2.14 Statistical analysis

All data analyses were conducted using a T-test, and the results are presented as means ± standard deviation (n ≥ 3, *p < 0.05, **p < 0.01 indicating significant differences).

## 3 Results and discussion

### 3.1 Characterization of nano-micelles

**FIGURE 1:**
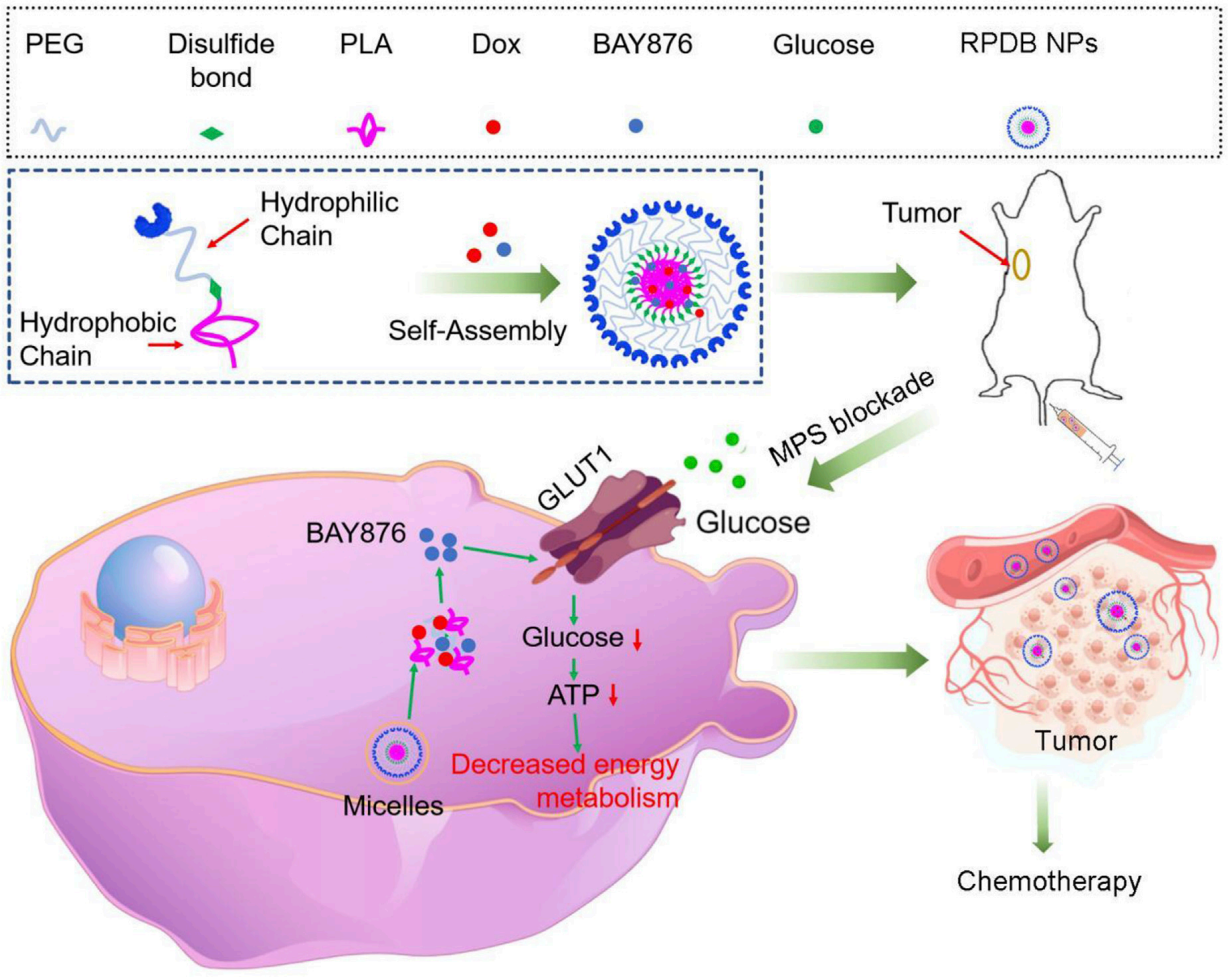
Schematics demonstrate the RPDB NPs blocking MPS for enhancing the antitumor efficacy. The intravenously injected nanoparticles were swallowed by macrophages, which release the GLUT1 inhibitor BAY876, reducing glucose entry into the cells. The phagocytic capacity of low-energy macrophages was downregulated, reducing the non-targeted effects and promoting drug aggregation in tumors.

The clinical application of nanomedicine is hindered by various biological obstacles, particularly the mononuclear phagocyte system (MPS), which leads to particle accumulation in macrophages such as Kupffer cells and reduces anti-tumor efficacy. Therefore, various anti-tumor strategies targeting the MPS system, especially liver macrophages, have been proposed, such as utilizing low immunogenic vectors to avoid the accelerated blood clearance (ABC) effect, saturating macrophage, and even eliminating macrophage (Figure 1).

**FIGURE 2:**
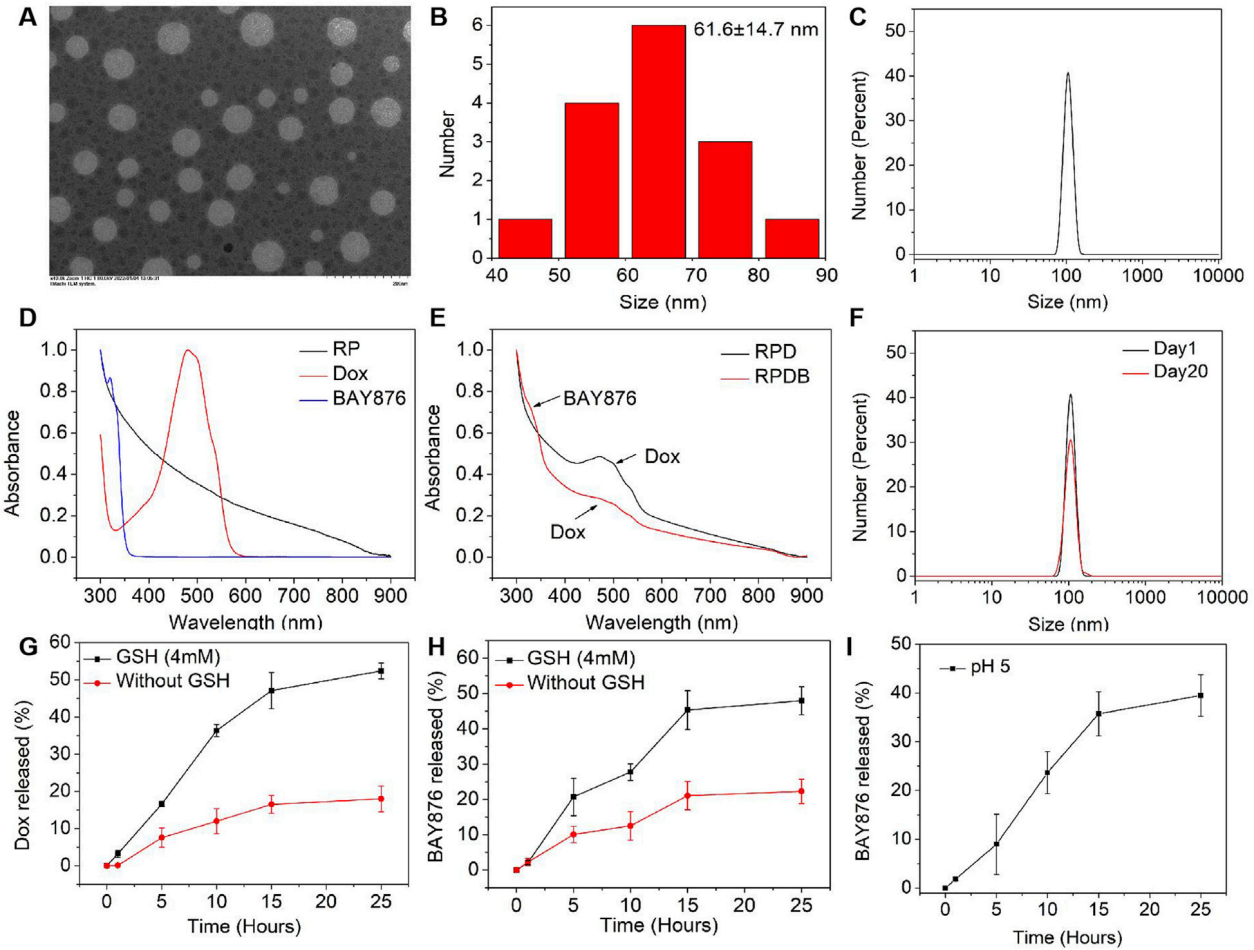
Characterization of RP-based nanoparticles. **(A)** TEM images of the RPDB NPs. **(B)** The size distribution of the RPDB NPs; **(C)** The mean hydrodynamic diameter distribution of RPDB NPs (105.7 ± 15.7 nm); **(D)** UV-vis spectra of BAY876, Dox and RP NPs; **(E)** UV-vis spectra of RPD NPs and RPDB NPs; **(F)** The stability of RPDB NPs was assessed by the mean hydrodynamic diameter distribution with FBS for 20 days. **(G)** The Dox release with and without GSH. **(H)** The BAY876 release with and without GSH. **(I)** The BAY876 release under different pH condition.

To further investigate the fundamental understanding of how glucose metabolism regulation affects the mononuclear phagocyte system (MPS) for enhancing tumor outcomes, nano-micelles were prepared in accordance with established protocols. In this study, drugs (Dox and BAY876 in chloroform) were encapsulated in PEG-PLA-based nano-micelles using the oil-in-water method. Subsequently, the nano-micelles were subjected to rigorous testing of their physical and chemical properties in order to ensure optimal drug loading efficacy. In Figure 2A, these nano-micelles were observed to have a spherical shape using transmission electron microscopy (TEM). The diameter distribution of the nano-micelles exhibited normality, with a mean value of approximately 61.6 ± 14.7 nm (Figure 2B). To further investigate size of nano-micelles, dynamic light scattering was utilized to characterize their mean hydrodynamic diameter (105.7 ± 15.7 nm, as illustrated in Figure 2C), which was consistent with the above data. For the formulation of nano-micelle complexes, the UV-vis spectrum analysis revealed that Dox and BAY876 exhibited strong absorption peaks at 480 nm and 320 nm, respectively (Figure 2D). After the synthesis of nano-micelles, the absorbance of these produces at 480 nm and 320 nm was detected, as shown in Figure2E. The encapsulation rate of Dox was 82.53% in RPD NPs and 55.12% in RPDB NPs. Similarly, the encapsulation rate of BAY876 was also evaluated to be 51.87% in RPDB NPs. Additionally, the biological efficacy of nano-micelles as an antitumor agent is hindered by their instability in blood. Therefore, we simulated the blood environment by incubating the sample in fetal bovine serum (FBS) at 37°C for a duration of 20 days. The hydrodynamic diameter mean did not exhibit significant alterations in Figure 2F when compared to Figure 2C. As previously reported, glutathione (GSH) can reduce disulfide bonds in tumor cells to form sulfhydryl groups, which not only depletes GSH but also facilitates drug release from PEG-S-S-PLA nano-micelles. Therefore, nano-micelles containing disulfide bonds were immersed in a 4 mM glutathione solution for varying durations to evaluate drug release. Compared to the absence of glutathione, it was observed that the release rate was significantly accelerated, with Dox and BAY876 releasing up to 45.3% and 47.1%, respectively, within 15 h (as depicted in Figures 2G, H). Considering the phagocytosis of macrophages, the release behavior of BAY876 was further explored under acidic conditions. As depicted in Figure 2I, the release of BAY876 from nano-micelles reached up to 35.7% within a time frame of 15 h, providing evidence for the potential inhibition of glucose transports in macrophages.

### 3.2 Inhibition of energy metabolism in macrophages

**FIGURE 3:**
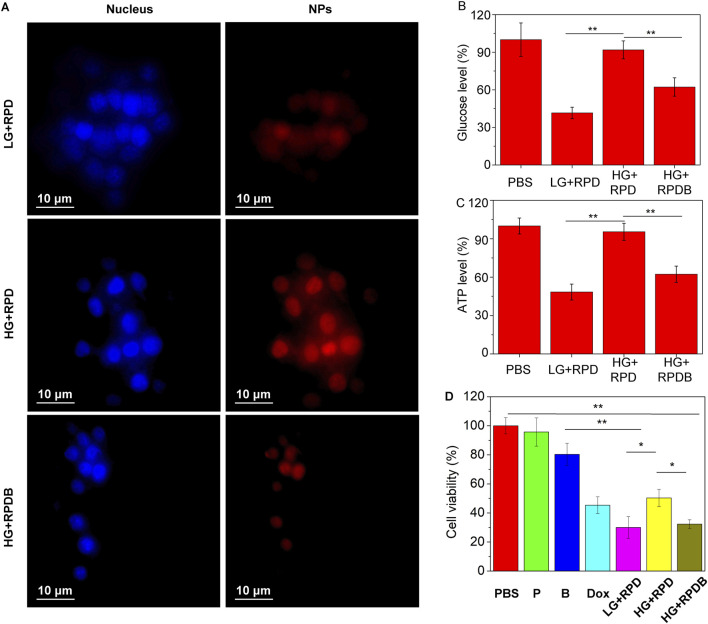
Evaluate the impact on energy metabolism inhibition in macrophages RAW264.7 cells. **(A)** Phagocytosis of nanoparticles by macrophages with different glucose concentrations. **(B)** The ratio of the glucose levels in nano-micelle-treated macrophages to those in the PBS group (n = 3, *p < 0.05, **p < 0.01). **(C)** The ratio of ATP levels between macrophages treated with nano-micelles and the PBS group (n = 3, *p < 0.05, **p < 0.01). The PBS group was incubated with high glucose (HG) DMEM. **(D)** The MTT assay was conducted for RAW264.7 cells viability, indicative of cytotoxicity. The PBS group was incubated with high glucose (HG) DMEM. The group LG + RPD represents the phagocytosis of RPD nanoparticles by macrophages under low glucose (LG) DMEM medium. Group HG + RPD represents the phagocytosis of RPD nanoparticles by macrophages under high glucose (HG) DMEM medium. Group HG + RPDB represents the phagocytosis of RPDB nanoparticles by macrophages under high glucose (HG) DMEM medium. Group P represents the addition of polymers alone; Group B represents the addition of BAY867 alone. (n = 3, *p < 0.05, **p < 0.01).

According to prior research, only 0.7% of ID nanoparticles were found to accumulate in tumors, primarily due to macrophage phagocytosis. Therefore, the regulation of macrophages has emerged as a promising strategy for cancer therapy and is receiving increasing attention. As a proof of concept, properties of constructing nano-micelles based on the energy metabolism of macrophages were verified here *in vitro*. As depicted in Figure 3A, macrophage RAW264.7 cells cultured in high glucose medium exhibited stronger fluorescence intensity compared to those cultured in low glucose medium, indicating the regulatory effect of glucose on macrophage endocytosis. However, incubation with the GLUT1 inhibitor BAY876 resulted in weaker fluorescence intensity of macrophages under high glucose conditions, indicating that the inhibitor reduced glucose uptake and subsequently minimized nanoparticle endocytosis. To further demonstrate this point, the glucose levels in macrophages from each group were assessed. The results indicated that the glucose level in group RPDB under high glucose conditions was significantly lower than that of group RPD, approaching levels observed in cells cultured with low glucose medium (as depicted in Figure 3B). Similarly, under high glucose conditions, group RPDB displayed lower ATP levels compared to group RPD, which is consistent with the glucose level trend (as shown in Figures 3B, C). At low glucose concentration, the cell survival rate was lower than that of group Dox. Similarly, under the same glucose concentration, the group RPDB with GULT1 inhibitor (BAY876) had lower vitality than that without GULT1 inhibitor, indicating that energy metabolism had an inhibitory effect on the viability of macrophages (as shown in Figure 3D). The decrease of glucose consumption leads to the slowing of aerobic respiration process and the increase of intracellular oxygen partial pressure, which may affect macrophage polarization and ROS production. Together with the aforementioned results, it was indicated that the effect of MPS was regulated by glucose metabolism for enhancing tumor outcomes.

### 3.3 *In vitro* antitumor evaluation

**FIGURE 4:**
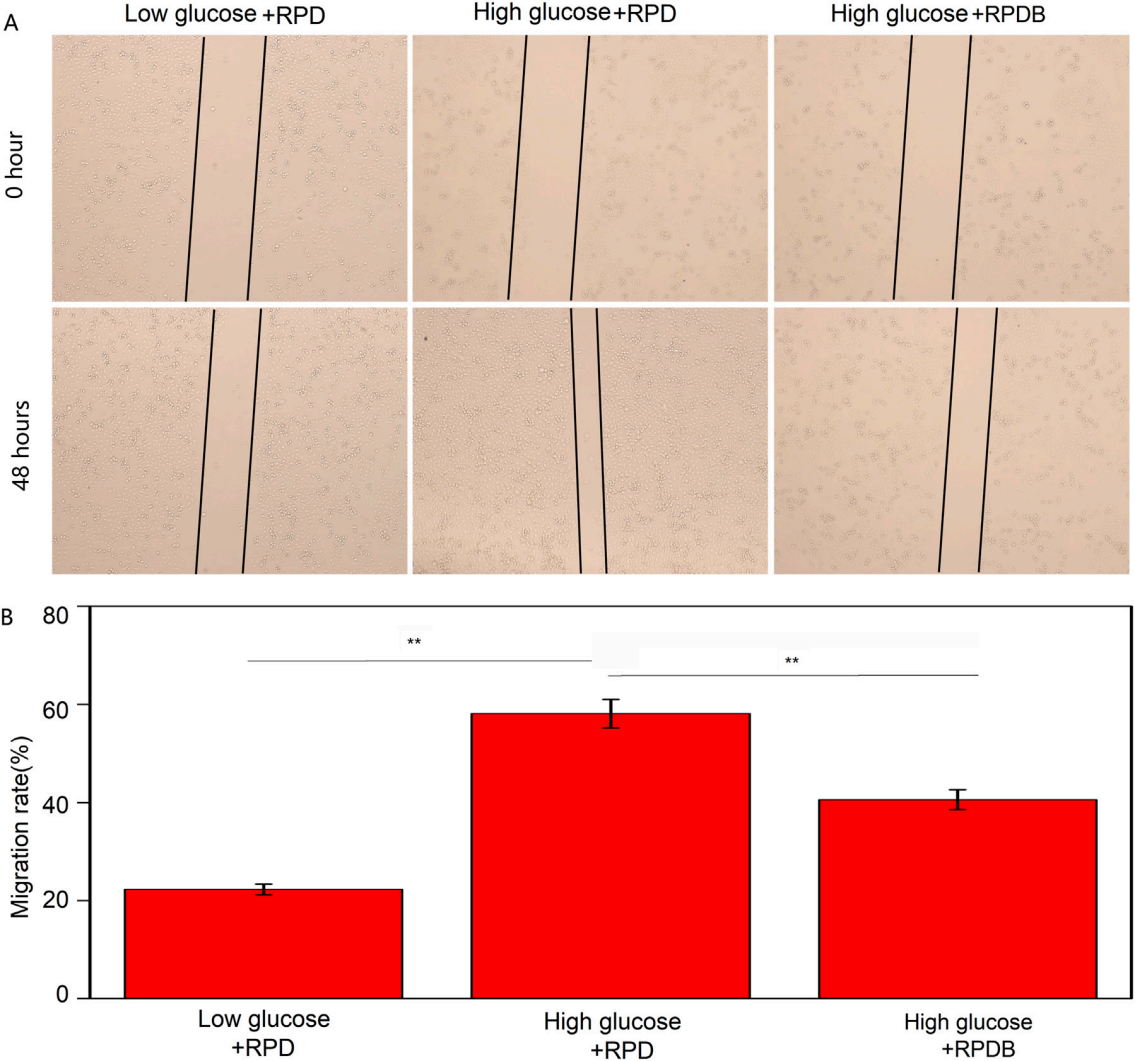
The migratory behavior was assessed by the scratch assay. **(A)** The impact of nano-micelle treatment on the migratory behavior of RAW264.7 cells was assessed within 0 and 48 h. **(B)** The migration rate was determined by administrating various nano-micelles.

Minimizing macrophage phagocytosis of inhibitor-loaded nano-micelles could reduce the MPS effect and prolong circulation time of blood for tumor therapy. Subsequently, the cytotoxicity of particles on tumor cells was evaluated *in vitro*, as displayed in Supplementary Figure S1A. The polymers demonstrated biocompatibility with 4T1 cells (as shown in Supplementary Figure S1A). Surprisingly, Dox-loaded polymers exhibited potent cytotoxicity surpassing that of injected Dox alone and BAY876. After 24 h of treatment, PDB NPs and RPDB NPs exhibited higher inhibition rates than other groups, indicating the GLUT1 inhibitor BAY876 blocked the glucose transport pathway. Among them, the RPDB NPs showed the higher inhibitory efficiency, which could be attributed to the expression of integrin on the cell membrane of 4T1. In Supplementary Figure S1B, cells were co-incubated with PDB NPs and RPDB NPs respectively, followed by staining with Hoechst33342. The fluorescent signal of RGD-modified nanoparticles was found to be stronger than that of PDB NPs, indicating the occurrence of RGD-mediated endocytosis process. This process facilitated the accumulation of nano-micelles in tumors, leading to glutathione (GSH) elimination and inhibition of energy metabolism. The downregulation of GSH levels in the polymer-treated groups, as demonstrated in Supplementary Figure S1C, was likely attributed to the presence of disulfide bonds within the polymer. Previous reports have shown that the levels of GSH in tumors are overexpressed than that in normal cells, and a decrease in GSH levels can enhance ferroptosis within tumors by blocking the synthesis of the lipid repair enzyme glutathione peroxidase 4 (GPX4) (Xiao et al., 2011; Yang et al., 2014). Similarly, BAY876, a GLUT1 inhibitor, possesses excellent properties for regulating glucose uptake. Since glucose is the fundamental source for cell metabolism, tumor cells rely on the Warburg effect and thus consume large amounts of it. When PDB NPs and RPDB NPs were introduced, inhibitor BAY876 was released to block the GLUT1, thereby reducing glucose levels in cell. Therefore, the PDB NPs and RPDB NPs groups exhibited a significant decrease in glucose levels compared to the PBS and RPD NPs groups, as illustrated in Supplementary Figure S1D. Meanwhile, both PDB NPs and RPDB NPs demonstrated lower ATP levels than the other groups in Supplementary Figure S1E, which is consistent with the observations presented in Supplementary Figure S1D. Supplementary Figure S1F shows that RPDB has an inhibition effect on RAW264.7 cells, but there is no significant difference from group Dox; Group BAY876 showed the same toxicity as group P, basically had no effect on cells. In addition, the scratch assay was conducted to further assess the correlation between glucose and macrophage migration. As shown in Figures 4A, B, the migration rate of the RPDB group in low glucose medium was slower, similar to that of the RPD group in low glucose medium, compared to the RPD group in high glucose medium.

### 3.4 Distribution of nanoparticles *in vivo*

**FIGURE 5:**
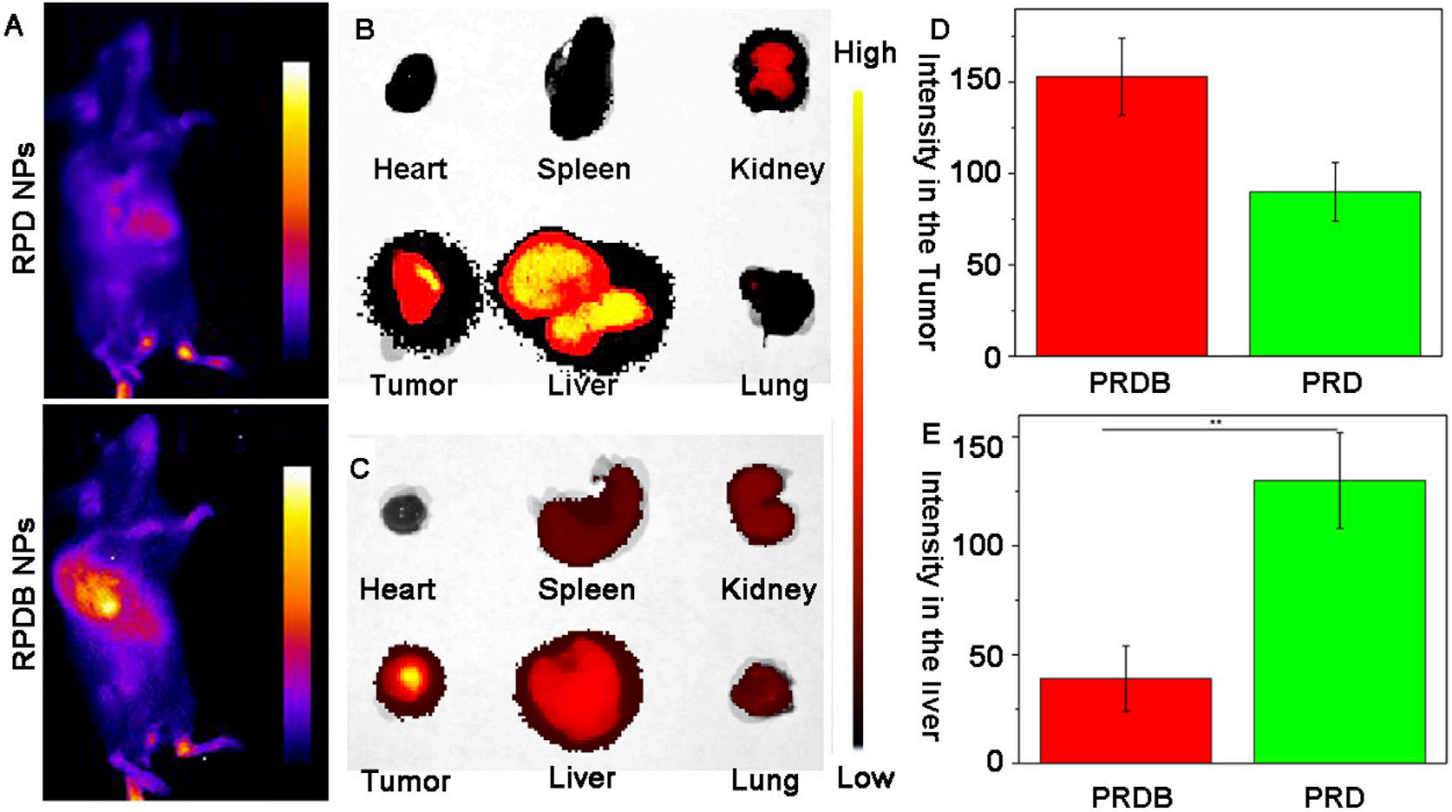
To assess the spatial distribution of fluorescently labeled nano-micelles in mice. **(A)** The fluorescence imaging of mice following intravenous injection *in vivo*. Blue indicates low fluorescence and yellow indicates high fluorescence; **(B)** The fluorescence images of major organs in group RPD NPs were collected; **(C)** The fluorescence images of major organs in group RPDB NPs were collected; **(D)** The fluorescence intensity of the tumor in mouse was semi-quantified using ImageJ software; **(E)** The fluorescence intensity of the liver in mouse was semi-quantified using ImageJ software.

As previously reported, the majority of delivery systems encounter biological barriers that result in drug accumulation within the liver, particularly in macrophages, leading to a reduction in drug concentration at tumor sites. To monitor the *in vivo* distribution of nano-micelles, RPDB NPs were modified with Cy5.5 fluorescent dye. Fluorescence signals were detected in the tumors of mice injected with both Cy5.5-modified nanoparticles (RPD NPs and RPDB NPs), as shown in Figure 5A and Supplementary Figure S2. However, RPD NPs exhibited a stronger fluorescence signal in the liver than RPDB NPs did, indicating that reducing energy metabolism of mononuclear phagocyte system-associated organs helped minimize drug accumulation in the liver. Within 24 h of injection, the fluorescence signal of RPDB NPs in the liver was weaker than RPD NPs, as shown in Figures 5B, C. Calculation showed that the tumor fluorescence intensity of the RPDB NPs group was 70% higher compared to that of the RPD NPs group, as depicted in Figure 5D. What’s more, the liver fluorescence intensity in the RPDB group was lower, about 40% of that in the RPD group (in Figure 5E). Therefore, the downregulation of macrophage energy metabolism through tailor-made nano-micelle complexes reduces the MPS effect and facilitates drug accumulation in tumors.

### 3.5 *In vivo* antitumor efficacy

**FIGURE 6:**
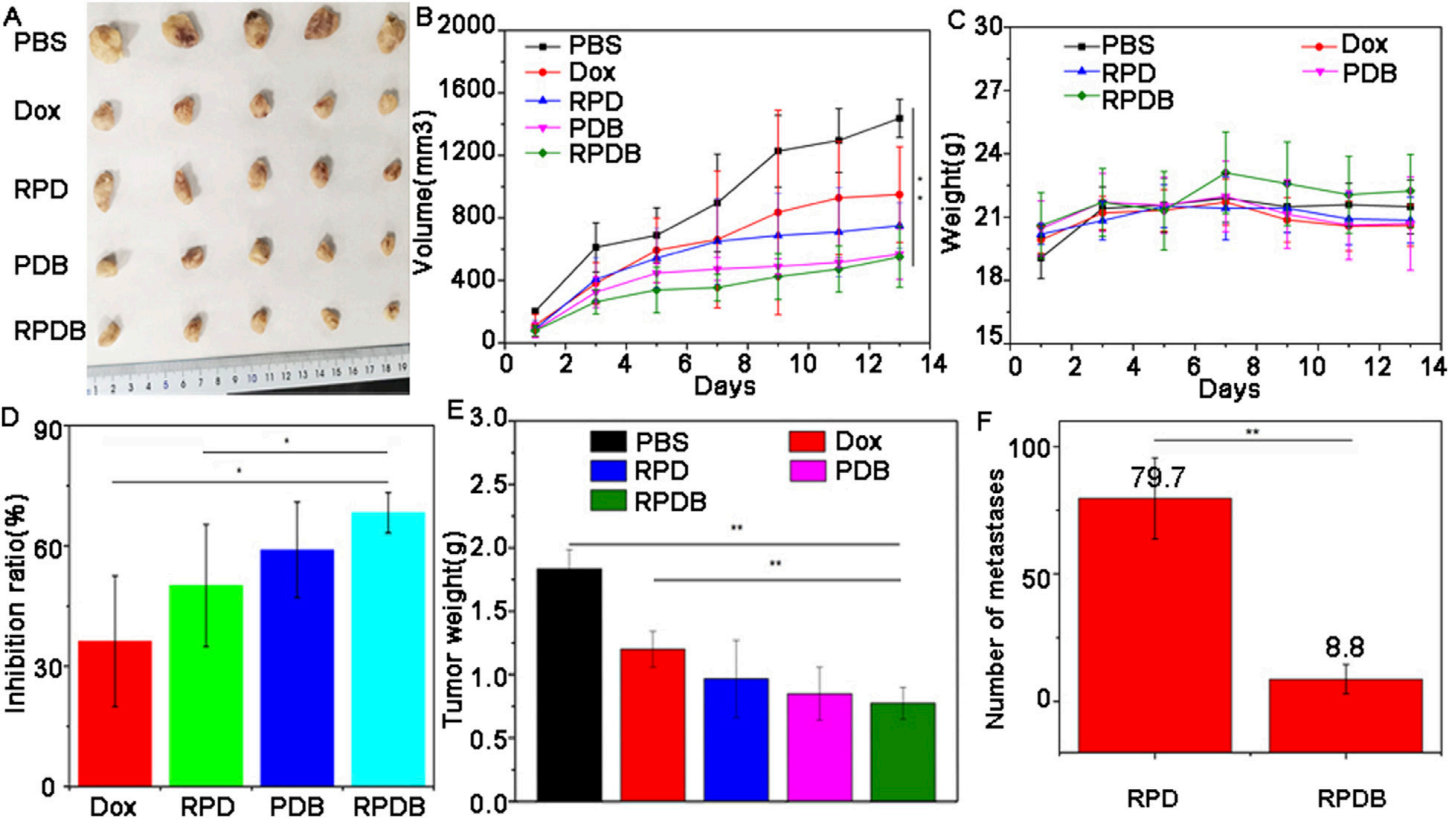
The *in vivo* antitumor efficacy of nano-micelles upon intravenous administration was evaluated. **(A)** The neoplasms were excised from the axillary region of the mouse forelimb on day 13 through surgical intervention; **(B)** The volume of tumor in mice injected with nano-micelles were meticulously measured for 13 days; **(C)** The weight trajectory of mice bearing tumors were meticulously recorded during the treatment period; **(D)** Inhibition rates of tumor growth across different groups of administration; **(E)** Tumor weights were meticulously recorded on day 13; **(F)** The number of metastases in mice of injected RPDB NPs and injected RPD NPs, and all data were presented as the mean ± standard deviation (n = 5, *p < 0.05, **p < 0.01). Number of metastases (Group RPD: 79.7 ± 15.9; Group RPDB: 8.8 ± 5.7).

Encouraged by distribution evaluation *in vivo*, these nano-micelles were further administrated intravenously into 4T1-tumor-bearing mice to assess antitumor outcomes. Similar to the assessment of cells toxicity, the experimental grouping and results were shown in Figure 6. After 13 days post-treatment, tumors in mice treated with PBS grew rapidly, while those in the other administration groups were significantly inhibited. These representative images of tumor have been captured and are shown in Figure 6A. The tumor volume was measured and calculated every 2 days over a period of 13 days, as illustrated in Figure 6B. In order to improve therapeutic efficiency, RGD was used to construct delivery systems targeting tumors. Evidently, the control group exhibited a significantly higher average tumor volume compared to the other groups, which is consistent with Figure 6A. Both PDB NPs group and RPDB NPs group exhibited excellent antitumor effects, as evidenced by the mean volume was significantly lower than that of the Dox and RPD groups. The difference in tumor inhibition effect between RPDB NPs and RPD NPs was significant, possibly due to the MPS effect being minimized by the GLUT1 inhibitor. Furthermore, body weight served as the most straightforward indicator for assessing drug toxicity. The absence of significant weight loss in mice during the treatment period, as shown in Figure 6C, indicates that these samples have low toxicity. Additionally, both the PDB NPs and RPDB NPs exhibited significantly higher tumor suppression ratios compared to the Dox and RPD NPs, with rates of 59.1% and 68.3%, respectively, versus 36.2% and 50.1%, as shown in Figure 6D. The inhibitory effects were further validated by the tumor weight presented in Figure 6E, which was consistent with both the tumor volume (Figure 6B) and inhibition rate (Figure 6D). As shown in Supplementary Figure S3, the living cell group (P1) had the most metastases in the lung, followed by the dead cell group and the living cell group (P3), and the dead cell group (P2) had no metastases. These results suggest that factors inhibit metastasis in the P3 group. Subsequently, RPD NPs and RPDB NPs were injected into group P3 respectively, and the results were shown in Figure 6F. RPDB NPs group had a significant lower metastases than RPD group, suggesting that RPDB NPs had an effect on inhibiting tumor metastasis.

### 3.6 Pathological analysis

**FIGURE 7:**
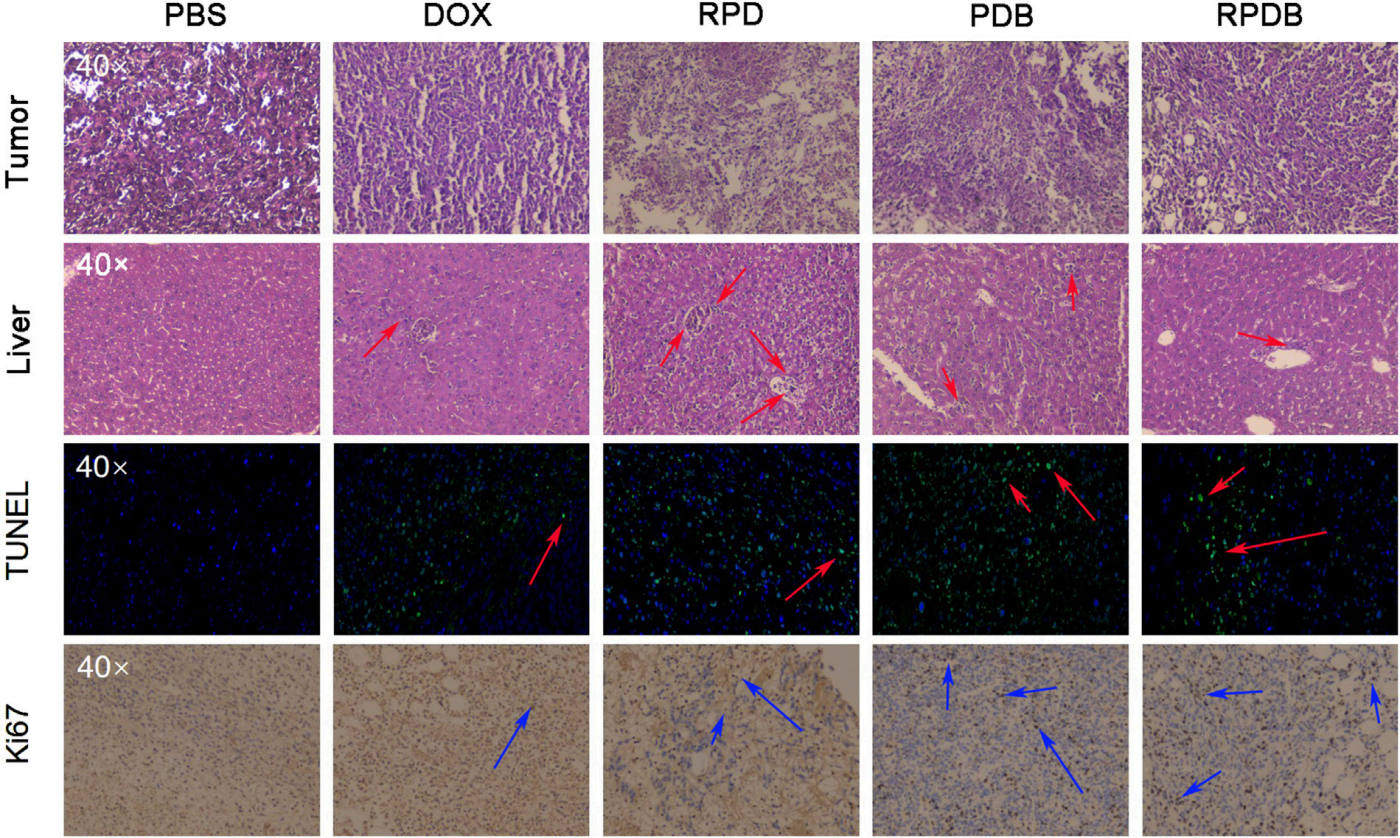
Thorough analysis was conducted on the liver and tumor pathological sections of mice injected with nano-micelles (n = 3). In the histopathological section of liver tissue, the red arrows represent Kupffer cells. In TUNEL analysis of tumors, a red arrow indicates DNA fragmentation, which is indicative of cell apoptosis in tumors. Ki67 serves as an indicator of tumor proliferation, with the blue arrow indicating positive areas.

Considering the excellent antitumor outcomes of nano-micelles based on macrophage energy metabolism, mice were euthanized to collect liver and tumor samples for pathological analysis. As shown in Figure 7, neither PDB NPs nor RPDB NPs showed a significant increase in Kupffer cells in H&E sections of mouse livers, while RPD NPs exhibited more Kupffer cells in hepatic sinusoids than other groups did, indicating that BAY876 contributed to the ability of nano-micelles to escape the RES effect. Meanwhile, the tumor sections of the PBS group also co-stained with hematoxylin and eosin, but did not present significant necrotic areas. However, in the treatment groups, damage areas were more pronounced, particularly in the PDB NPs and RPDB NPs groups. Based on TUNEL results, the RPDB NP-treated group exhibited a higher number of fluorescent spots indicating DNA breakage within tumors. Such DNA breakage often leads to cell death. Furthermore, the RPDB NPs group exhibited significantly stronger inhibition of cell proliferation compared to the other groups. The Ki-67 staining results revealed that the PBS group displayed a significantly higher proliferation factor compared to the other groups. During the treatment period, both the PDB NPs and RPDB NPs groups showed negligible proliferation of tumor cells. Therefore, pathological analysis suggests that anti-tumor strategies based on energy metabolism could minimize macrophage phagocytosis for tumor inhibition *in vivo*.

### 3.7 *In vivo* biosafety evaluation

**FIGURE 8:**
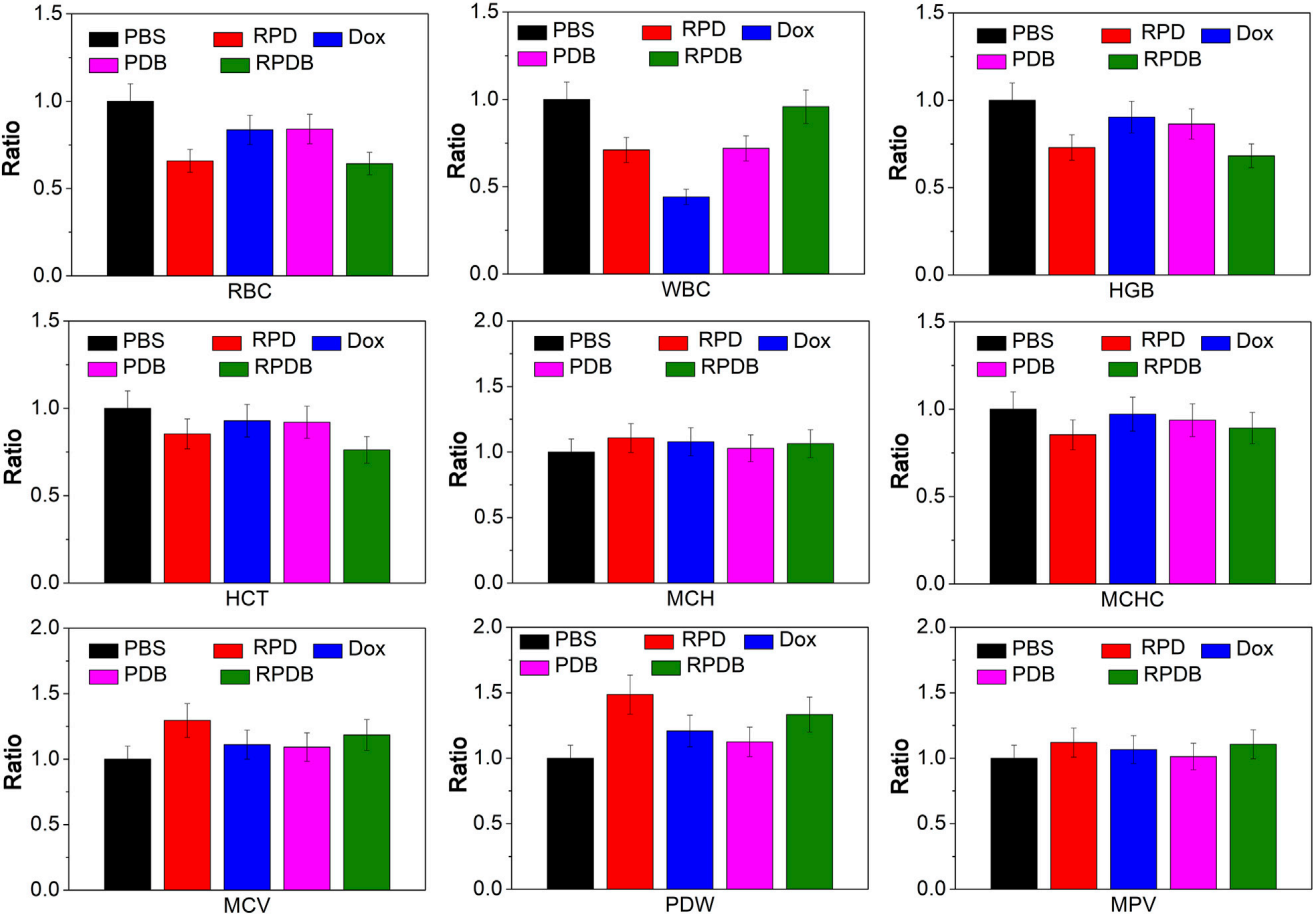
The safety of nano-micelles *in vivo* was preliminarily evaluated by analyzing blood routine.

Macrophages in the liver play a crucial role in the immune system. The biosafety of anti-tumor strategies aimed at down-regulating macrophage energy metabolism is further discussed concerning routine inspection and H&E staining. As shown in Figure 8, blood routine results indicate a significant decline in leukocytes for group Dox, consistent with previous reports of acute toxicity. However, nano-micelle complexes RPDB did not induce a drop in leukocytes. Comparing other indicators (HCT, MCH, MCHC, MCV, PDW, and MPV), no significant differences were seen between these groups. RPDB has a weak effect on RBC and HGB, which may be caused by inhibiting GLUT1 of erythrocytes. H&E staining was employed for further *in vivo* safety evaluation of nano-micelles, as shown in Supplementary Figure S4. Negligible inflammation was observed in the heart, lungs, spleen, and kidneys of both the PDB and RPDB groups compared to the PBS group. The results indicate that both the PDB and RPDB groups demonstrate exceptional biocompatibility, thus holding significant potential for future antitumor applications.

## 4 Conclusion

Nanoparticles were plagued by various biological obstacles in clinical practice, especially the RES effect of macrophages, which led to a large amount of nanoparticle aggregation in the liver. In this study, nano-micelles complexes were successfully constructed by encapsulating BAY876 and Dox with polymer PEG-SS-PLA. These complexes exhibited excellent inhibitory effects on macrophage energy metabolism to minimize MPS effect. *In vivo*, the fluorescence signal of RPDB NPs was significantly weaker than that of RPD NPs in the liver, indicating that regulating macrophage energy metabolism helped to regulate drug distribution. Similarly, results from pathological sections also showed that there were more macrophages (Kupffer cells) in the groups without inhibitor BAY 867 compared to those with inhibitor. Meanwhile, these nano-platforms have shown significant antitumor outcomes *in vitro* and vivo. Compared with other MPS inhibition strategies, inhibition of energy metabolism did not eliminate macrophages and was beneficial to the recovery of immune function. As expected, nano-micelles based on the energy metabolism of macrophages could reduce the MPS effect for nanoparticles and improve antitumor outcomes, lighting up an alternative strategy in clinical application.

## Data Availability

The original contributions presented in the study are included in the article/Supplementary Material, further inquiries can be directed to the corresponding author.
