# Supplementary material for: Inhibition of energy metabolism in macrophages to block MPS for enhancing the chemotherapy efficacy
Source: Front Bioeng Biotechnol. 2025 Apr 4;13:1549101. doi: 10.3389/fbioe.2025.1549101 (PMC12006136; doi:10.3389/fbioe.2025.1549101)
Supplement: Supplementary file 1 [file DataSheet1.pdf]

Inhibition of energy metabolism in macrophages to block MPS for enhancing the chemotherapy efficacy

Li Bin[1,2], Linlin Huang[1], Aiyu Chen[1], Yinyi Yang[1], Yanmei Zheng[1], Hanwen Zhang[1], Qinfang Zhang[1], Jiahui Zheng[1], Meiting Qiu[1],Xiajin Li[1], Yangbo Tan[1]*

1. Department of Medical College, Guangxi University of Science and Technology, Liuzhou 545005, China.

2. Laboratory animal Center，Liuzhou People's Hospital，Liuzhou, Guangxi 545000, China.

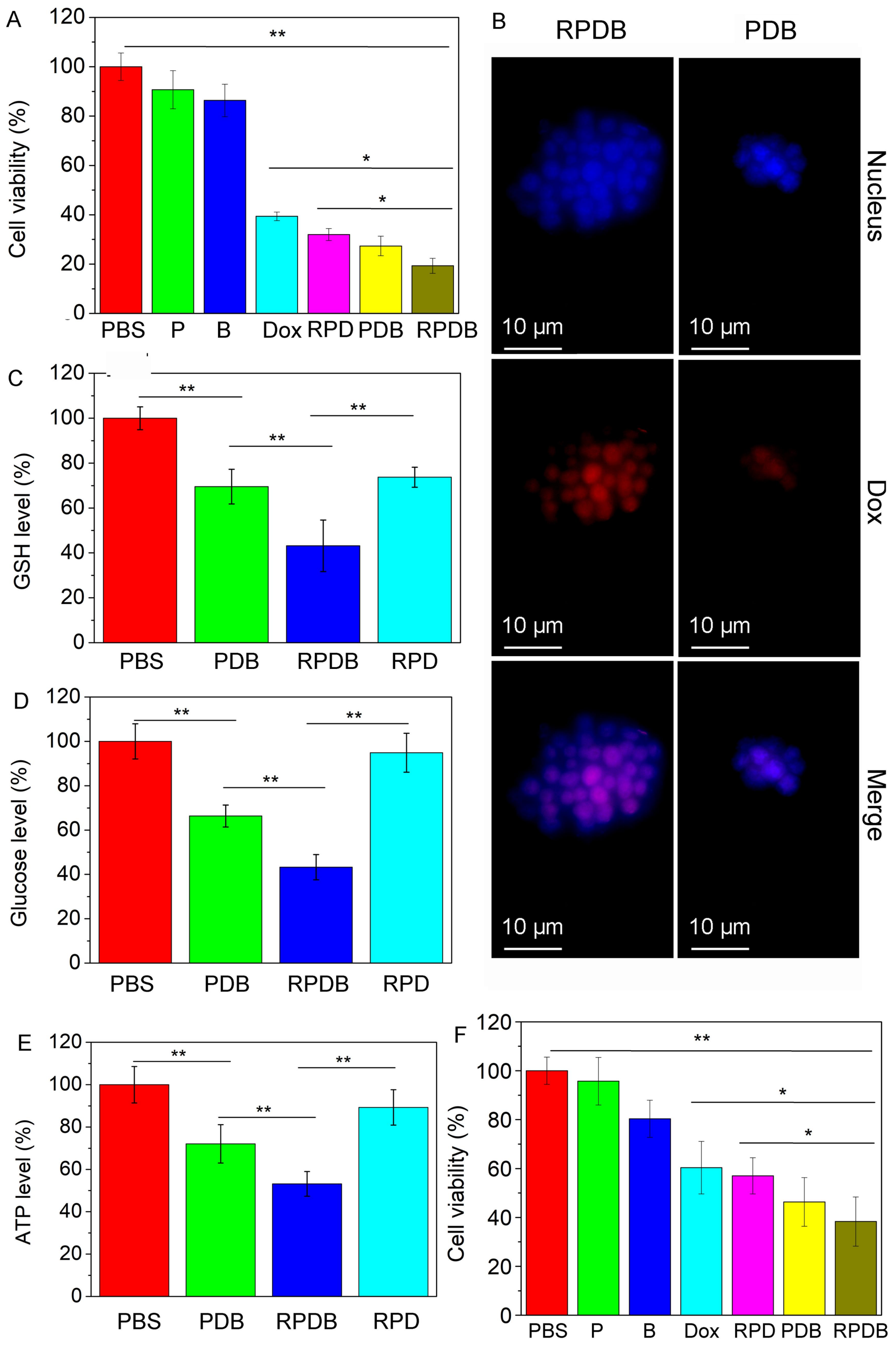

Supplementary Figure 1. The assessment of cellular cytotoxicity and endocytosis was conducted in vitro. (A) The MTT assay was conducted for 4T1 cell viability, indicative of cytotoxicity; (B) The

endocytosis in 4T1 cells was visualized in vitro using a microscope. Scale bar: 10 µm; (C) Changes in intracellular GSH levels of 4T1 cells under various treatments; (D) Changes in intracellular glucose levels of 4T1 cells under various treatments; (E) Changes in intracellular ATP levels of 4T1 cells under various treatments. (F) The assessment of cellular cytotoxicity for RAW264.7 cells was conducted in vitro.(n = 3, *p < 0.05, **p < 0.01).

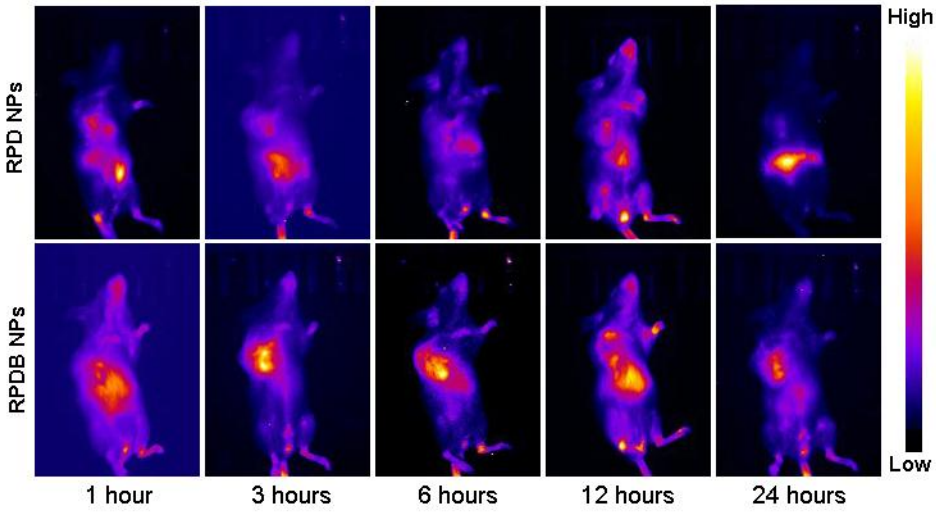

Supplementary Figure 2. To assess the spatial distribution of fluorescently labeled nano-micelles in mice.

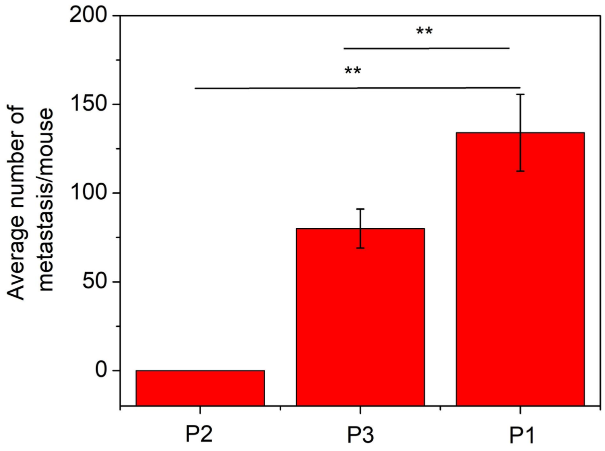

Supplementary Figure 3. The number of metastases in mice.P1group (Each mouse was injected with 5×10^6 live cells), P2 group (Each mouse was injected with 5×10^6 dead cells), P3 (Each mouse was

injected with 5×10^6 dead cells and 5×10^6 live cells). ($n = 5$, $*p < 0.05$, $**p < 0.01$).

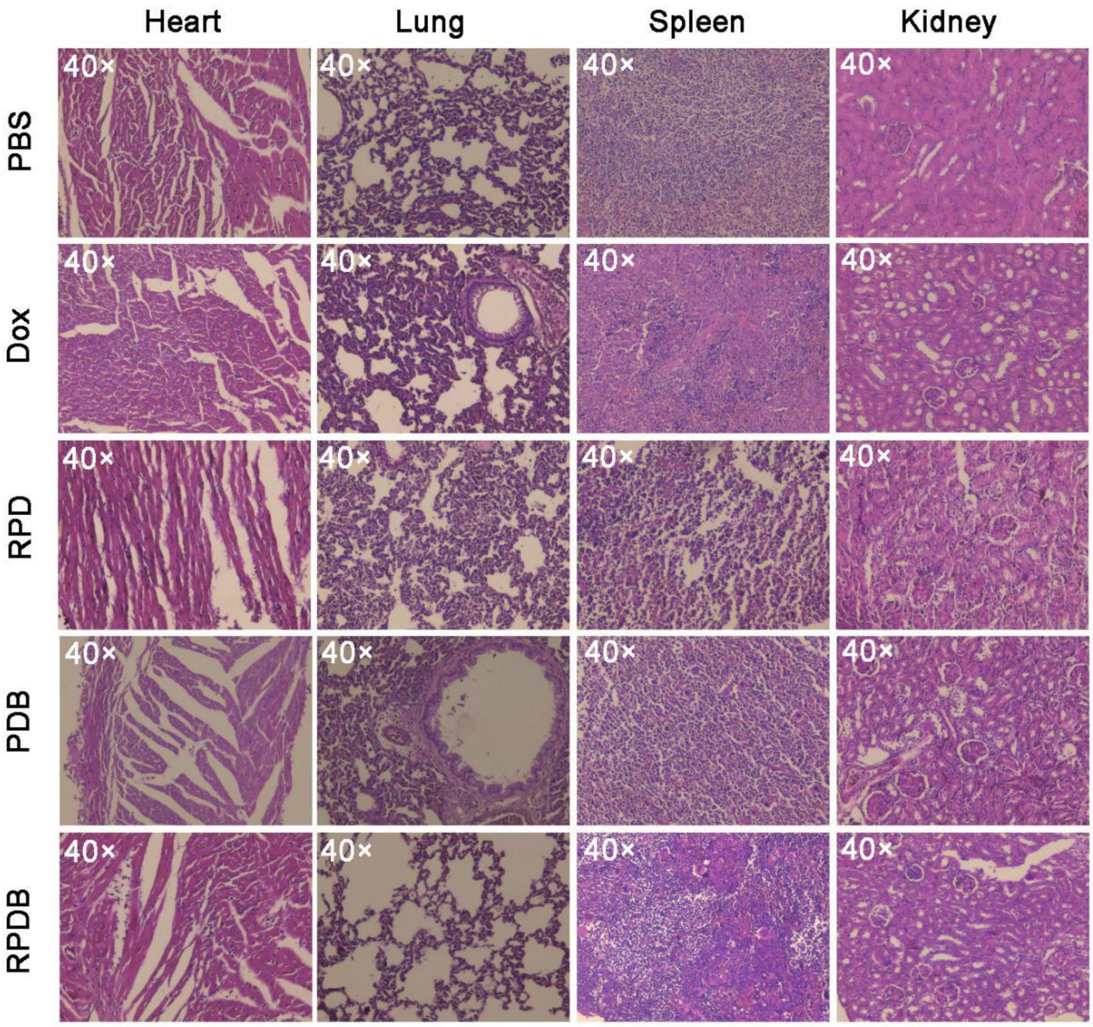

Supplementary Figure 4. The primary safety assessment was conducted by histologically examining major organs from treated mice using H&E staining.
